# Review of Control Techniques in Microinverters

**DOI:** 10.3390/s21196486

**Published:** 2021-09-28

**Authors:** Diego Rojas, Javier Muñoz, Marco Rivera, Jaime Rohten

**Affiliations:** 1 Engineering Systems Doctoral Program, Faculty of Engineering, University of Talca, 3340000 Curicó, Chile; dnprojas@gmail.com (D.R.); marcoriv@utalca.cl (M.R.); 2 Department of Electrical and Electronic Engineering, Universidad Del Bío-Bío, Avenida Collao 1202, 4051381 Concepción, Chile; jrohten@ubiobio.cl

**Keywords:** control strategies, DC-DC converter, DC-AC converter, microinverter, maximum power point tracking, photovoltaic

## Abstract

The use of renewable energies sources is taking great importance due to the high demand for electricity and the decrease in the use of fossil fuels worldwide. In this context, electricity generation through photovoltaic panels is gaining a lot of interest due to the reduction in installation costs and the rapid advance of the development of new technologies. To minimize or reduce the negative impact of partial shading or mismatches of photovoltaic panels, many researchers have proposed four configurations that depend on the power ranges and the application. The microinverter is a promising solution in photovoltaic systems, due to its high efficiency of Maximum Power Point Tracking and high flexibility. However, there are several challenges to improve microinverter’s reliability and conversion efficiency that depend on the proper control design and the power converter design. This paper presents a review of different control strategies in microinverters for different applications. The control strategies are described and compared based on stability, dynamic response, topologies, and control objectives. One of the most important results showed that there is little research regarding the stability and robustness analysis of the reviewed control strategies.

## 1. Introduction

Electricity generation systems through photovoltaic panels are becoming increasingly important within renewable energies sources, since the costs associated with photovoltaic panels have decreased and the efficiency of power converters have increased [1]. Due to environmental policies and the growth in electricity demand, the use of photovoltaic panels has grown worldwide, today having a total installed capacity of 623 GW approximately [2]. However, when implementing or selecting an electrical generation system, high robustness should be considered in the face of voltage variations, current or power outage, high reliability (appropriate waveforms for both voltage and current and supply of electrical energy in all moment), and an adequate power capacity for the design requirements, in order to obtain a good electrical production performance. These systems generate many transient, due to the variation in the solar radiation, and therefore a non-continuous supply, leading to power quality issues [3,4]. A significant part of the photovoltaic installations are the power conditioning system, also known as power converters, which transform the electrical power, generated by the photovoltaic panels, into a signal suitable for use. In order to mitigate these problems, or reduce the negative effects, different configurations have been proposed, such as the string, multistring, central, and ac module, where the main differences are given by the power range. For example, a string configuration operates between 1 kW and 10 kW (residential application), a multistring configuration operates between 10 kW and 30 kW (residential or commercial application), a central configuration operates from 30 kW (large-scale photovoltaic plants), and a ac module configuration or microinverter operates at a maximum of 500 W (small-scale systems) [5]. Moreover, they are also differentiated by the series and parallel combination of the photovoltaic panels and their respective connection to the power converters.

The central configuration has arrived to the market with the greatest impact, having a market-share of almost 95%, due to its high efficiency (close to 98%) and the high demand for electricity generation [6]. On the other hand, a microinverter is a configuration which allows for the integration of photovoltaic solar energy, where each photovoltaic module contains its own converter. They are also known as ac modules or integrated module inverters, because they are small and operate in a low power range [1]. The advantages of this configuration are high Maximum Power Point Tracking (MPPT) efficiency, ease of installation, flexibility, being modular, better amortization of the initial investment [7], ease of monitoring and detecting faults [1], applications in small power, and it can be installed in complex structures with different orientations and it is not necessary to incorporate bypass diode [7].

However, up to now the microinverter’s configuration has a low market entry, having a market-share of less than 10%, low operating power ranges, and low reliability. Other shortcomings are low conversion efficiency (up to 96.5% [1,6]), higher cost per watt, and in the absence of boost converter it requires a bulky power transform and it requires a high boost to pump up the voltage to the grid level.

Due to the rapid development of new power semiconductors, microinverters are an emerging and promising solution to mitigate the partial shading and dirt-effect problems. Thanks to the recent advances, it will be possible to increase the ranges of power and conversion efficiency. In addition, to ensure a safe, reliable, and efficient energy conversion from photovoltaic generation systems, it is very important to consider the adequate design of the control of the power converters, as well as the topology configuration. Hence, control strategies are important to regulate the different voltage and current levels for the requirements of different applications, with the aim of increasing the reliability of the microinverter. Therefore, this paper will be focused on different types of control strategies applied in microinverters for a range of purposes.

## 2. Microinverter

Microinverters can be classified into four categories [8,9], such as: one-stage topology without galvanic isolation; two-stage topology without galvanic isolation; one-stage topology with galvanic isolation; and two-stage topology with galvanic isolation.

**Figure 1:**
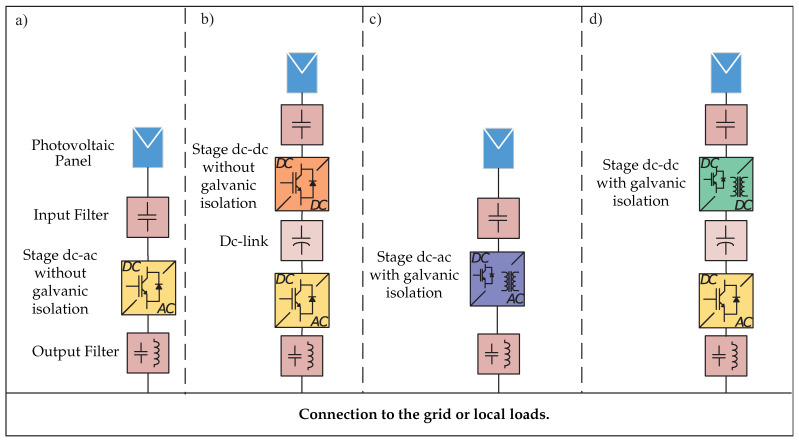
Classification of microinverters. (**a**) One-stage topology without galvanic isolation. (**b**) Two-stage topology with galvanic isolation. (**c**) One-stage topology with galvanic isolation. (**d**) Two-stage topology with galvanic isolation.

Figure 1 shows the configuration of each category. In a two-stage topology, it consists of a dc-dc converter that performs the MPPT, and the dc-ac converter has the responsibility of controlling the dc-link and the control of the grid current or properly controlling the output voltage in island mode. In terms of control, the two-stage topology is simple, and the dc-dc converter also extends the operation of the photovoltaic system, leading to a decrease in overall efficiency [6]. One-stage topologies are introduced to reduce power losses and reduce the total system volume [6].

On the other hand, microinverters can also be classified by the incorporation of galvanic isolation depending on the electrical policies of each country, as well as the needs of photovoltaic installation. The incorporation of a transformer allows for the isolation of the photovoltaic generation stage and the consumption stage or the grid, with the aim that if panels fail, it does not have direct impact on the grid or on local loads. In addition, the transformer allows reaching high levels of voltage, which is required for integration to the grid; however, it leads to a reduction in efficiency reduction and an increase in the microinverter’s volume [6,8,10].

However, there are certain challenges in positioning microinverters as an attractive alternative on the market [6,8], such as: to increase reliability and lifespan due to sensitivity to the temperature of the electrolytic capacitors; to increase conversion efficiency considering cutting-edge semiconductors and development of new high-gain converter topologies; and to increase the functionality of the microinverters, adding some other tasks such as reactive power support and power supply at all times.

### Design Challenges

Despite having several advantages, in the market they are not the device that users buy the most due to low power ranges, low reliability, and low conversion efficiency compared to the other configurations [6]. Other disadvantages are that they have a single function, aimed to make the conversion of electrical energy necessary for their use or inject into the grid. Microinverters are usually equipped with bulky, low-reliability capacitors, which have a high rate of failure [7]. On the other hand, because solar energy is intermittent, a storage system with high capacity and a fast charging/discharging is required. One of the possible solutions to improve reliability is the incorporation of a hybrid storage (supercapacitor and battery) with the aim of increasing power density and energy density. Moreover, the cost of rechargeable energy storage has decreased drastically in recent years due to technological advances due to higher penetration of distributed Renewable Energy Sources. Additionally, if this battery/ultracapacitor hybrid energy storage system is embedded in the PV micro-inverters, the problem of reliability that electrolytic-capacitor-based micro-inverters have can be overcome, together with the filtering of the power ripple, and it will allow an additional ancillary service as backup for the power grid acting as a distributed Uninterruptible Power Supply, providing a distributed inertia to the utility grid.

On the other hand, the proposed solution to increase power capacities, improve efficiency, and reduce the size of the microinverter is the implementation of semiconductors based on Gallium-Nitride and Silicon-Carbide since they are characterized by being of high efficiency, high power density, high frequency operation, and they decrease the size of the converter [11]. As these materials allow much higher switching speeds, it implies smaller passive components, increasing thus the power density of micro-inverters but also taking into account that the circuit parasitics and the associated electromagnetic interference may be reduced to unprecedentedly low levels, demanding new approaches of fully integrated assemblies comprising power devices, gate drives, filters, and control functions.

## 3. Control Strategies

This section presents a review of control strategies applied to microinverters and it is organized depending on the control application: grid connected, islanding mode (off-grid), reactive power compensation, photovoltaic system including energy storage, and multiple operation mode or multiple functions.

### 3.1. Grid Connected

**Figure 2:**
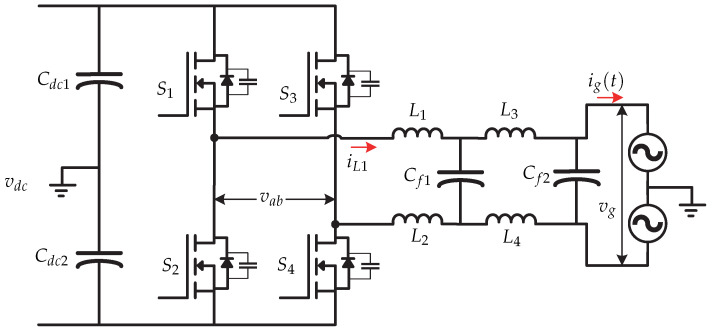
Microinverter’s topology proposed in [12].

**Figure 3:**
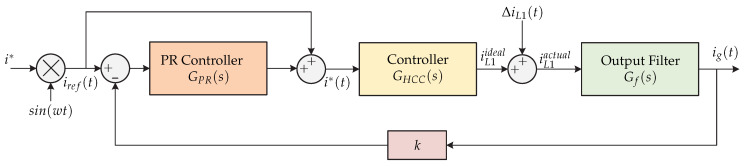
Control strategy proposed in [12].

The reference [12] presents a hybrid hysteresis current control $G_{HCC}\left(s\right)$ and a low-frequency harmonic mitigation strategy based on a PR (Proportional-Resonant) control $G_{PR}\left(s\right)$. Tthe microinverter’s topology is a full-bridge inverter for the PV system (Figure 2). The main objective is to reduce the switching losses and achieve an optimized grid current. Figure 3 shows the proposed control strategy. Due to a wide hysteresis band for smooth switching, low output inductance, and digital controller sampling time, the error between the reference current $i^{*}\left(t\right)$ and the average inductor output current still results from many low frequency harmonics, so the current grid $i_{g}\left(t\right)$ must be sensed and compared to a reference. The low frequency error can be mitigated by the PR controller and the power quality of the electrical grid could be improved.

**Figure 4:**
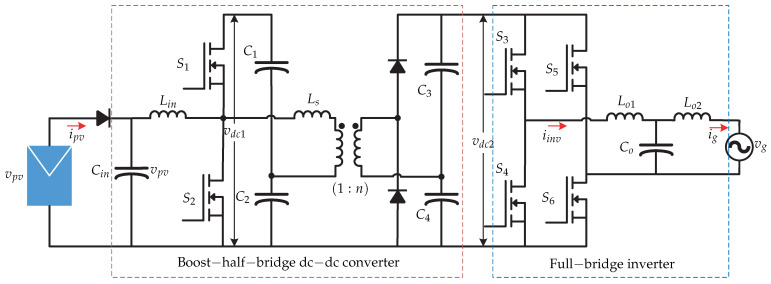
Microinverter’s topology proposed in [13,14].

**Figure 5:**
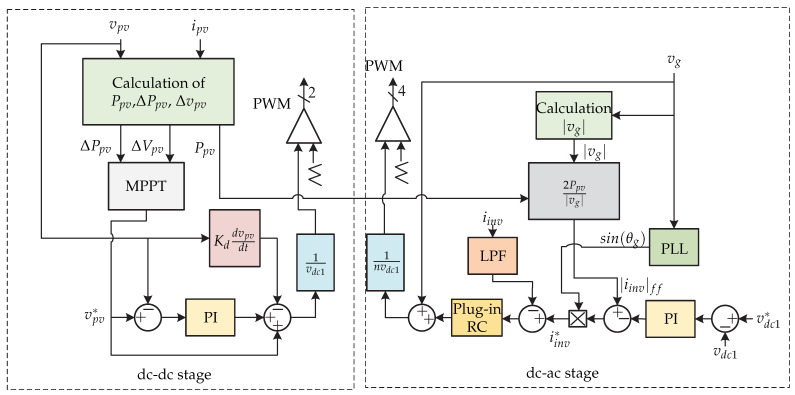
Control strategy proposed in [13,14].

In [13,14] a two-stage microinverter consisting of a boost-half-bridge converter connected to an full-bridge inverter is presented (Figure 4). The control strategy is presented in Figure 5. The dc-dc converter is in charge of the maximum power point tracking and generates the voltage reference $v_{pv}^{*}$, which is compared with the voltage of the photovoltaic panel $v_{pv}$. The voltage error is minimized by a Proportional-Integral controller (PI), and the result is summed up to the panel photovoltaic voltage reference and compared with the derivative of the panel voltage. The result is the modulating pattern, which is compared to a triangular carrier signal that generates the switching signal of the dc-dc converter. In the dc-ac stage there is a double control loop, where the external voltage loop is handled by a PI controller that generates the grid peak current reference. Then, it is compared with the norm of the inverter current $|i_{inv}{|}_{ff}$ and then the result is multiplied by a sinusoidal signal $sin(\theta_{g})$ whose angle is obtained from a Phase Locked Loop (PLL). This inverter current reference $i_{inv}^{*}$ is compared with the sensed inverter current $i_{inv}$ whose result is minimized by a repetitive controller (Plug-in RC) to generate the switching signals of the full-bridge inverter. To achieve fast dynamic responses in both grid current as well as dc-link voltage, a feed-forward current reference is added to the control strategy.

**Figure 6:**
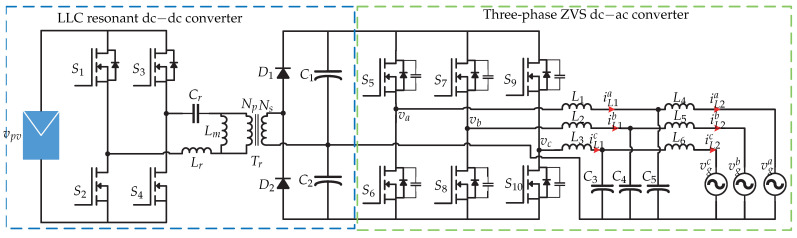
Microinverter’s topology proposed in [15].

**Figure 7:**
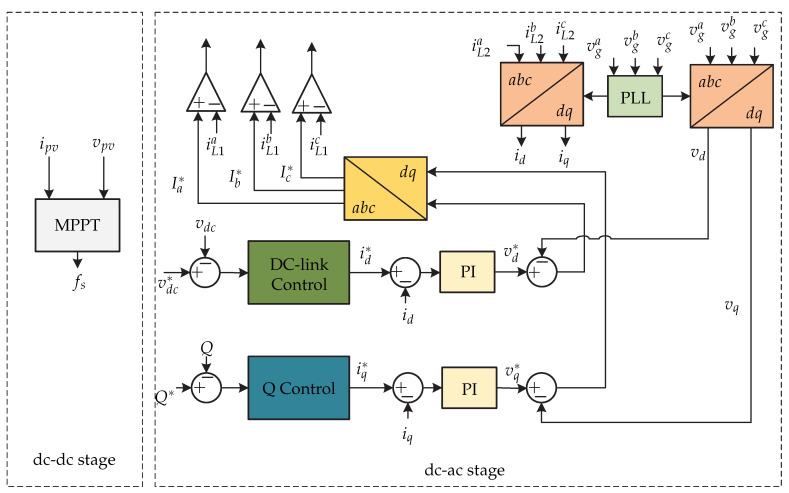
Control strategy proposed in [15,16,17].

The references [15,16] present a dual-stage microinverter, where in the first stage is an LLC resonant dc-dc converter and in the second stage a three-phase zero-voltage-switching (ZVS) dc-ac converter (Figure 6). The variables to be controlled in the microinverter are the inductor currents ($i_{L2}^{a},i_{L2}^{b}$ and $i_{L2}^{c}$) used as a filter, the dc-link voltage $v_{dc}$ and the currents injected into the grid ($i^{a},i^{b},i^{c}$). Figure 7 presents the control strategy. The objective of the dc-dc converter is to track the maximum power point by means of an MPPT algorithm, whose function is to define the switching signal $f_{s}$ based on the power of the photovoltaic panel. In the dc-ac stage, the proposed control strategy is that of a triple loop controller. In order to balance the power of the photovoltaic panel and the power of the electrical network, it is necessary to have a constant dc-link voltage. This is done by comparing the dc-link voltage $v_{dc}$ with a reference $v_{dc}^{*}$ and the result is controlled by a dc-link controller that generates the current reference in the *d*-frame and is compared with the current $i_{d}$ obtained by the dq transformation. The result is controlled by a PI controller that generates the voltage reference in the *d*-frame $v_{d}^{*}$ which is sum to the grid voltage. The Q control is not explained in [15] because reference current in *q*-frame is considered zero. On the other hand, the reference current $i_{q}^{*}$ compared with the grid current in the *q*-frame is controlled by the mean of a PI controller to generate the reference grid voltage in the *q*-frame $v_{q}^{*}$ which is which is the reference for the sensed grid voltage $v_{q}$. Then, the reference three-phase current ($I_{a}^{*},I_{b}^{*}$ and $I_{c}^{*}$), and then it is compared with the inductance currents ($i_{L1}^{a},i_{L1}^{b},i_{L1}^{c}$) in order to generate the switching sequence.

**Figure 8:**
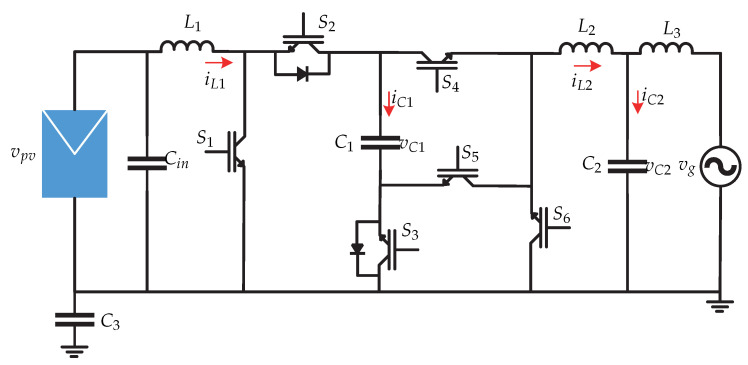
Microinverter’s topology proposed in [18].

**Figure 9:**
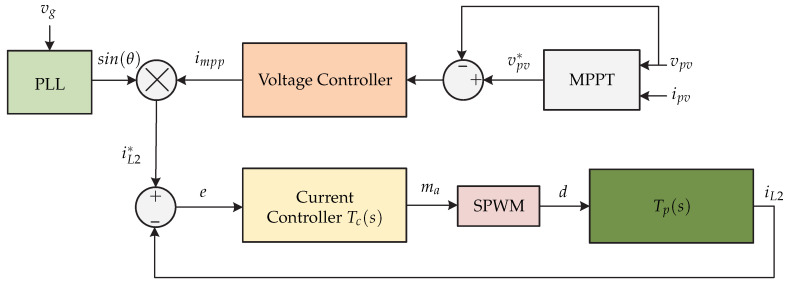
Control strategy proposed in [18].

In [18] a microinverter without galvanic isolation is presented and it consists of a topology derived from a non-inverted Cuk converter connected to an inverted Cuk converter (Figure 8). The proposed control strategy is presented in Figure 9. The control strategy consists of an MPPT algorithm that generates the reference voltage $v_{pv}^{*}$ that is compared with the voltage of the instantaneous photovoltaic panel $v_{pv}$. The result is processed by a PI voltage controller that generates the current for the photovoltaic panel at the maximum power point $i_{mpp}$. This signal is multiplied by the sinθ provided by a PLL to synchronize the signal with the grid voltage, obtaining the inductor current reference $i_{L2}^{*}$. This signal is compared with the sensed inductor current $i_{L2}$ and is processed by a PR type current controller to generate the modulator $m_{a}$. This modulating signal is necessary to generate the switching sequence by means of a SPWM.

**Figure 10:**
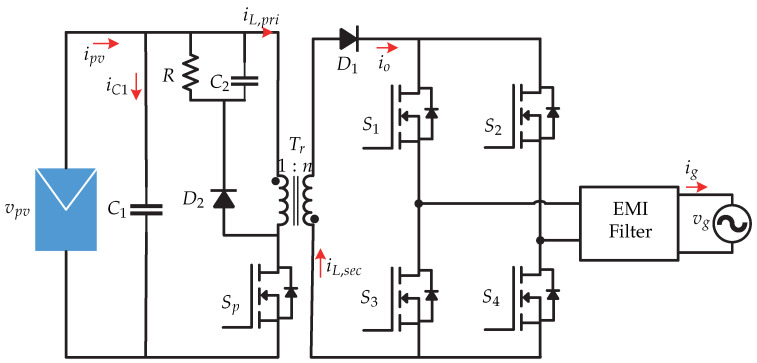
Microinverter’s topology proposed in [19,20].

**Figure 11:**
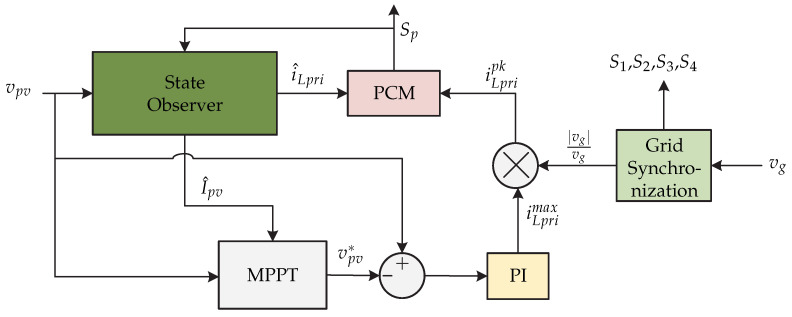
Control strategy proposed in [19,20].

In [19,20] a current sensorless control strategy is proposed for a dual stage microinverter. It consists of a flyback dc-dc converter connected to a voltage source inverter for the current injection to the electrical grid (Figure 10). The advantage of this proposal is the cost derived from current sensors by minimizing and reducing measurement noise introduced to the control algorithm. The control strategy is presented in Figure 11 and consists of an observer that estimates the inductance values to calculate the inductor current based on the comparison between the results of two calculations of the current. The state observer estimates the value of the magnetizing current ${\hat{i}}_{L,pri}$ and the current of the photovoltaic panel ${\hat{i}}_{pv}$. Then, the MPPT (Perturb and Observe) algorithm generates the photovoltaic panel voltage $v_{pv}^{*}$ reference which is compared to the sensed photovoltaic panel voltage $v_{pv}$. The result is processed by a PI controller that generates the maximum permissible magnetizing current $i_{L,pri}^{max}$ and is multiplied by the voltage of the synchronized network to generate the peak magnetization current $i_{L,pri}^{pk}$. Finally, a peak current control (PCM) is developed to generate the switching signal $S_{p}$ that depends on the peak magnetization current and the estimated magnetization current. It is worth mentioning that the proposed work only presents the control strategy for the dc-dc stage and an open loop for the inverter, leaving the control strategy for the dc-ac stage as future work.

**Figure 12:**
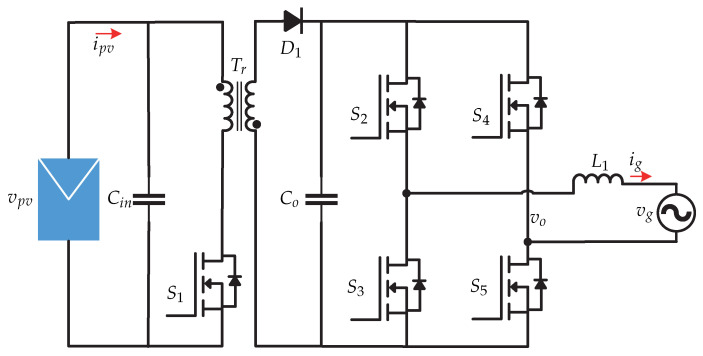
Microinverter’s topology proposed in [21,22,23,24].

**Figure 13:**
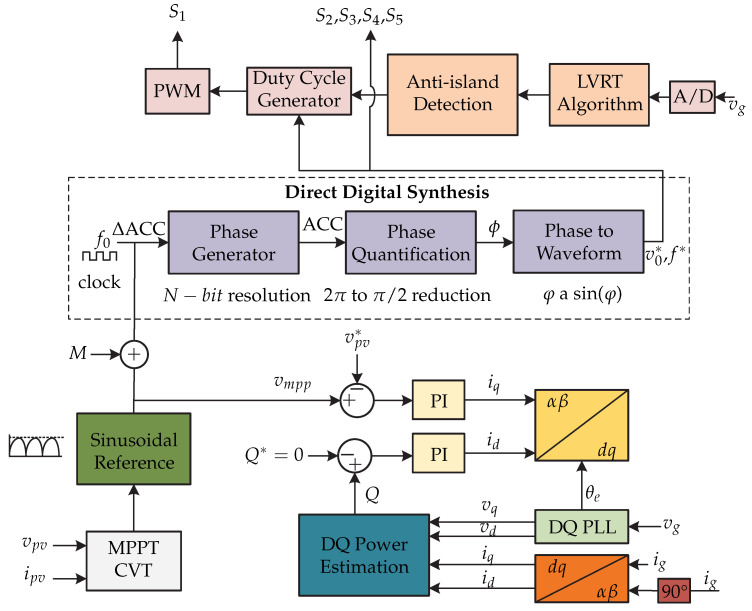
Control strategy proposed in [21,22,23,24].

In [21,22,23,24] a microinverter (Figure 12) based on a direct digital synthesis technique is presented for operation in grid-connected mode. This technique provides flexibility in implementation of various controls such as MPPT, PLL, anti-island, and low-voltage ride-though (LVRT). The control strategy is presented in Figure 13 and consists of the MPPT algorithm based on the constant voltage method (CVT) for the dc-dc stage, which requires sensing the voltage and current of the photovoltaic panel ($v_{pv}$ y $i_{pv}$). The MPPT algorithm generates the voltage at the maximum power point $V_{mpp}$ and it is compared with a reference voltage $v_{pv}^{*}$ and the error is processed by a PI controller that generates the *d* frame current ($i_{d}$). On the other hand, the grid voltage $v_{g}$ and the grid current $i_{s}$ are sensed to generate the currents and voltages in dq frame that are used by a dq power estimator. The output of the estimator is the Q reactive power which is compared with the reference reactive power $Q^{*}$. The error is processed by a PI controller that generates the current in *q* frame ($i_{q}$). Then, the dq frame currents are transformed to the αβ frame. In summary, the *d* component regulates the output voltage $v_{o}$ and the *q* component determines the reactive power to be injected into the grid.

**Figure 14:**
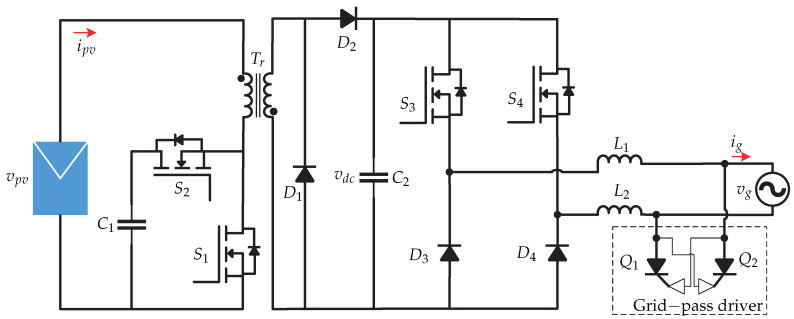
Microinverter’s topology proposed in [25].

**Figure 15:**
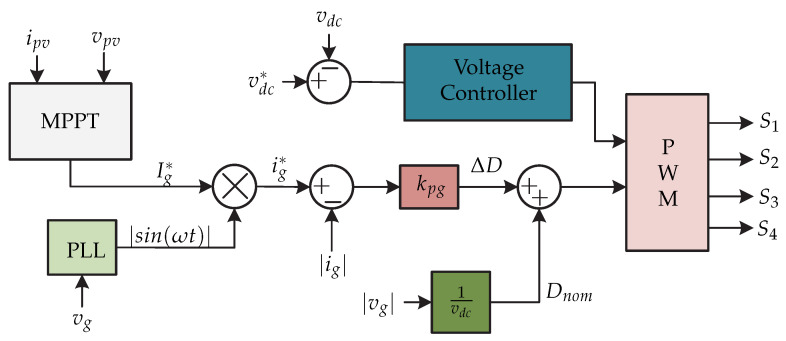
Control strategy proposed in [25].

In [25] a microinverter consisting of two stages is presented. The dc-dc stage consists of a flyback converter with an *active-clamp* circuit in the primary part of the transformer and a *series resonant voltage doubler* in the secondary part of the transformer (Figure 14). The *active-clamp* circuit allows for the operation of the switches in zero-voltage switching by limiting the voltage across the active power semiconductors and therefore reducing the losses. The control strategy is presented in Figure 15 which consists of the MPPT algorithm that generates the amplitude of the grid current reference $I_{g}^{*}$. This is multiplied by the signal obtained from the PLL in order to obtain the reference grid current $i_{g}^{*}$. Then this signal is compared to the grid current $|i_{g}|$ to generate the variation of the duty cycle ΔD by means of a proportional controller $k_{pg}$. The term $|v_{g}|/v_{dc}$ is the product of the applied feedback linearization, to decouple the variation of the duty cycle with rated duty cycle $D_{nom}$, in order to make the relationship between of the variation of the grid current $\Delta i_{g}$ and ΔD first order and linear. Furthermore, the voltage regulation is performed on the dc-link voltage $v_{dc}$ by means of a voltage controller. The signal generated by the voltage controller and the sum of the duty cycle variation and the rated duty cycle generate the switching signals for the dc-dc converter and the dc-ac converter ($S_{1},S_{2},S_{3},S_{4}$).

**Figure 16:**
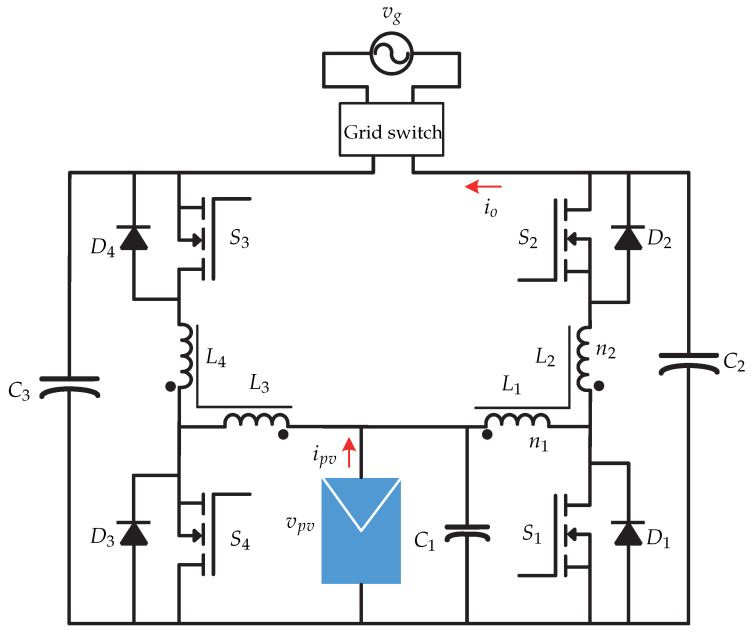
Microinverter’s topology proposed in [26].

**Figure 17:**
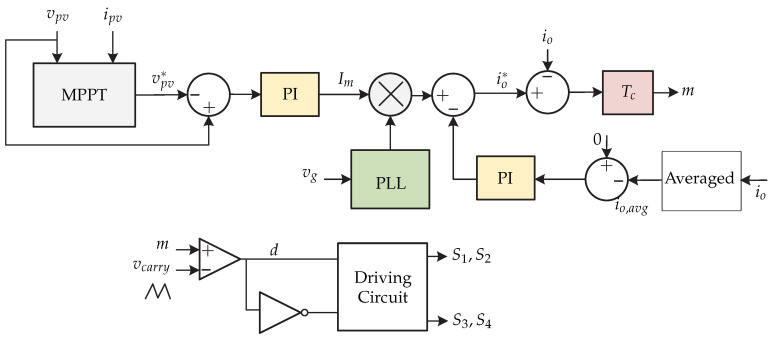
Control strategy proposed in [26].

In [26] a single stage dc-ac microinverter is presented consisting of a coupled-inductor double-boost inverter (Figure 16). The main characteristics are: simple structure, generation of an ac output in magnitude greater than the dc signal, small volume, and high efficiency. The control strategy is presented in Figure 17. The control consists of an MPPT P and O algorithm where the reference voltage of the photovoltaic panel $V_{pv}^{*}$ is obtained and it is compared with the photovoltaic panel voltage $v_{pv}$, whose error is processed by a PI voltage control to generate the current amplitude $I_{m}$. The amplitude is multiplied by a sinusoidal signal, whose angle is obtained from a PLL for synchronization with the grid, obtaining the reference of the output current $i_{o}^{*}$. The reference is compared with the output current $i_{o}$ and enters a current controller proposed in [26] and allows canceling the dc components of the inductor current, as well as canceling some poles and zeros of the proposed converter model.

**Figure 18:**
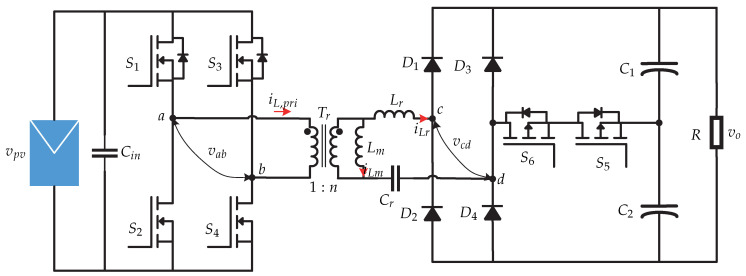
Microinverter’s topology proposed in [27,28].

**Figure 19:**
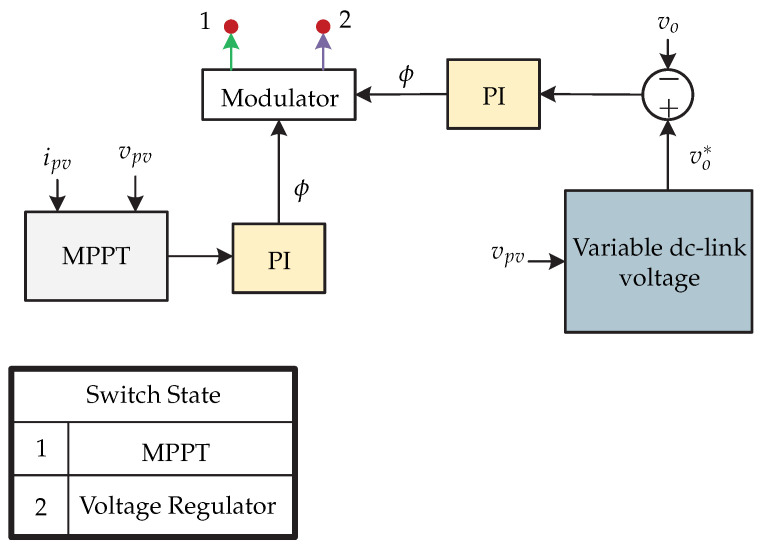
Control strategy proposed in [27].

In [27,28], the study of the dc-dc stage of a microinverter is presented, which is suitable for connecting it to a dc-ac stage and integrating it with the grid (Figure 18). A topology of a dual-mode rectifier (DMR) based series resonant dc-dc converter is proposed and its characteristics are: ability to operate in a wide variety of voltage inputs and high efficiency. The control strategy proposed for the dc-dc converter is presented in Figure 19 and its main characteristic is its variable dc-link voltage control, with the aim of reducing RMS (root mean square) currents, improving the efficiency of the microinverter. In addition, it is characterized by being a flexible control, since it has two modes of operation. For the case of the control of the MPPT, Mode 1 will be applied. The MPPT algorithm is executed and the voltage reference is compared with the voltage of the photovoltaic panel $v_{pv}$. Then the comparison result is processed by a PI control to get ϕ (inverter controls the dc-link). In Mode 2, the switch connects the dc source and disconnects the photovoltaic panel. Then, the variable control is executed which generates the reference voltage of the dc link $v_{o}^{*}$ and is compared with the output voltage $v_{o}$. Finally, the result of the comparison is processed by a PI control to obtain the value of ϕ. The switching sequence is generated by the modulator depending on the operating mode of the microinverter.

**Figure 20:**
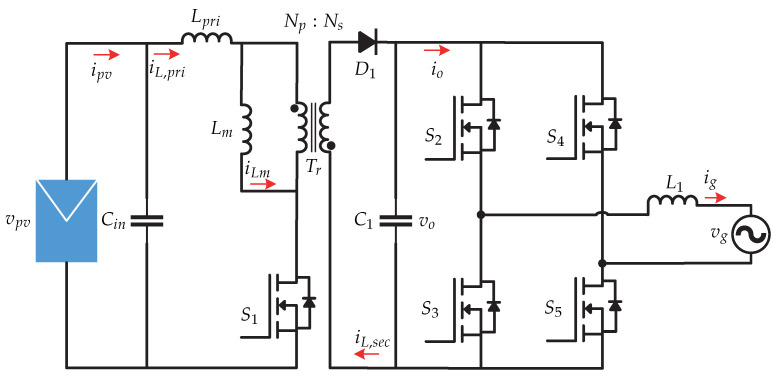
Microinverter’s topology proposed in [29].

**Figure 21:**
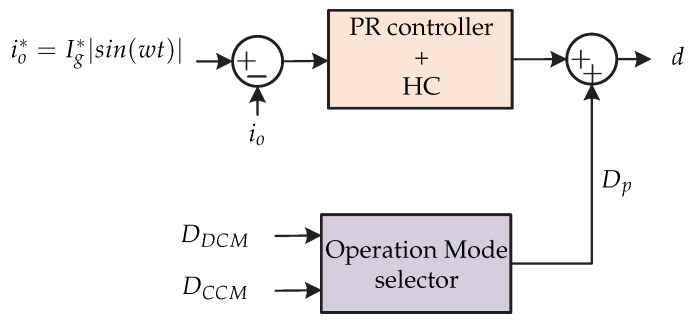
Control strategy proposed in [29].

In [29] a control strategy for a two-stage topology is presented. In the first stage, it contains a flyback dc-dc converter and in the second stage it contains a full-bridge inverter (Figure 20). A microinverter with hybrid mode is presented and consists of a control strategy that allows the system to operate in both continuous and discontinuous mode. The advantages of operating in hybrid mode is the stress reduction faced by the primary and secondary part of the transformer [29]. The control strategy is presented in Figure 21. The proportional-resonant controller (PR controller) plus the harmonic compensator (HC) provide a high gain in the fundamental frequency and harmonics, in order to improve the performance of the discontinuous mode and the stability of the continuous mode of the flyback converter. In addition, the hybrid duty cycle obtained by means of the operating mode selector allows for the elimination of disturbances and reduces the load on the feedback controller.

Other control techniques are as follows:

- The paper [30] presents a Flyback PV microinverter with analog and digital controller. The analog control consists of a precision rectifier circuit, a pulse width modulation comparator, and zero-crossing detector. The aim of digital control is to obtain the Maximum Power Point from the Photovoltaic module.
- The paper [31] presents a differential boost microinverter. The control technique consists of a MPPT-loop control, a second loop that synchronizes the grid current to the grid voltage, and a three-loop differential peak current control.
- The paper [32] presents a two stage microinverter with LLC resonant converter. The control technique consists of a MPPT based a fixed-frequency model predictive control and a PI control.
- The paper [33] presents a microinverter based a cascaded boost converter with a full bridge. The control technique consists in two sliding control alternatives (input current mode and pseudo-oscillating mode).
- The paper [34] presents a microinverter based a interleaved flyback with an unfolding H-bridge inverter. The control technique consists in a novel sliding mode control current controller.
- The paper [35,36] presents a interleaved flyback with two stage unfolding cycloconverter. The control strategy consists in a power-increment-aided incremental-conductance MPPT with constant-frequency variable-duty and a forward compensator.
- The paper [37] presents a dual-active-bridge (DAB) microinverter. The control strategy consists in simple closed-loop current control (PI controller).
- The paper [38] presents a switched capacitor buck-boost voltage source inverter (SC-BBVSI). The control strategy consists in a PI controller for dc-link voltage regulation and proportional-resonant (PR) controller for injected current regulation.
- The paper [39] presents a two stage microinverter. This consists of a high step-up Z source dc-dc converter with a full-bridge inverter. The control technique is similar to [13] (Figure 4).
- The paper [40] presents a two stage microinverter and it consists of boost dc-dc converter with a single-phase full-bridge inverter. The control strategy consists in non-linear control techniques based of the non-linear average model of microinverter.
- The paper [41] presents a boost inverter and it consists of two boost dc-dc converters connected in differential mode to the grid. The control technique consists of a PI control for power reference and a flatness-based control. In [42], a flatness-based control is also presented.
- The paper [43,44] presents two microinverter topologies. First, a interleaved flyback dc-dc converter with unfolding inverter is presented and then a push–pull dc-dc converter with unfolding inverter. The proposed control strategy consists in a simple PR controller to generate a sinusoidal current reference waveform and PI controller to generate a power reference.
- The paper [45] presents a two-stage microinverter and it consists in a step-up isolation dc-dc converter with half-bridge inverter. The control technique consists in a PI controller in order to reduce the third harmonic. Moreover, it consists in a feedforward control.
- The paper [46] presents a full-bridge inverter for microinverter application. The control technique consists in a sliding mode control of the output current.
- The paper [47] presents a quadratic boost dc-dc converter with full-bridge inverter. The control technique consists in a sliding mode control for dc-link voltage and grid current regulation. The paper [48] presents the above topology, but the control strategy is based on PI controllers.
- The paper [49] presents a multi-level single phase microinverter and its control strategy consists in a model predictive control to reduce the steady state error of the grid-injected current. Another control technique used in this microinverter is the PI controller with PR controller proposed in [50].
- The paper [51] presents a single stage boost inverter, composed by a two bidirectional boost dc-dc converter. The control strategy consists in a finite control set model predictive control algorithm with predictions of the system variables.
- The paper [52] presents a full-bridge converter cascaded to a boost converter with other full-bridge converter. The control technique consists in a PI controller for dc-link voltage regulation and a PR controller used in the current control loop.
- The paper [53] presents a T-type microinverter in boundary conduction mode. The control technique consists in a hybrid control based on the proposed voltage equalization and adaptive reverse current control method.
- The paper [54] presents a high-gain Z-source boost converter with H-bridge inverter. The control strategy consists of a PI controller to regulate the dc-link voltage and hysteresis current control to regulate the grid current.
- The paper [55] presents a resonant microinverter and its control strategy consists of different PI controllers.
- The paper [56] presents a microinverter based in a modified current source inverter. The control strategy consists in two PI controllers and dq transformation.
- The paper [57] presents a flyback dc-dc converter with line-frequency inverter. The control strategy consists in a inverse model with a single closed-loop PI controller.
- The paper [58] presents a boost-half-bridge dc-dc converter and full-bridge inverter. The control technique consists in a repetitive current controller based on fourth-order linear phase IIR filter. The repetitive current controller is used to reduce the total harmonic distortion and current regulation. There is a PI controller in the dc-dc stage.
- The paper [59] presents a flyback-based partial power dc-dc converter with a H-bridge inverter. The control strategy consists in a cascaded control loop (PI controllers) for dc-dc stage and a classical single-phase voltage oriented control algorithm for dc-ac stage.
- The paper [60] presents a coupled inductor based cúk dc-dc converter connected to the line frequency current unfolding stage. The control strategy is comprised of different PI controllers.
- The paper [61] presents a LLC dc-dc converter connected to a full-bridge inverter. The control strategy of dc-ac stage consists in a dead-beat scheme. The control strategy of dc-dc stage consists in a simple closed-loop PI control.

### 3.2. Island Mode

**Figure 22:**
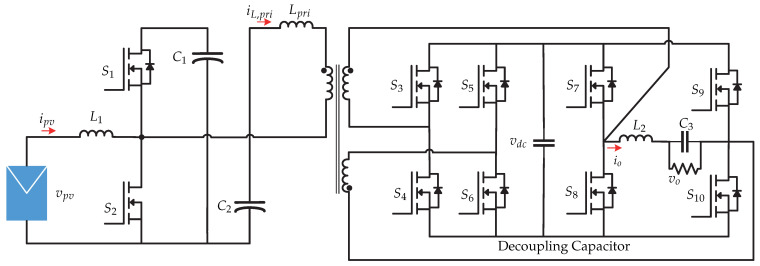
Microinverter’s topology proposed in [62].

**Figure 23:**
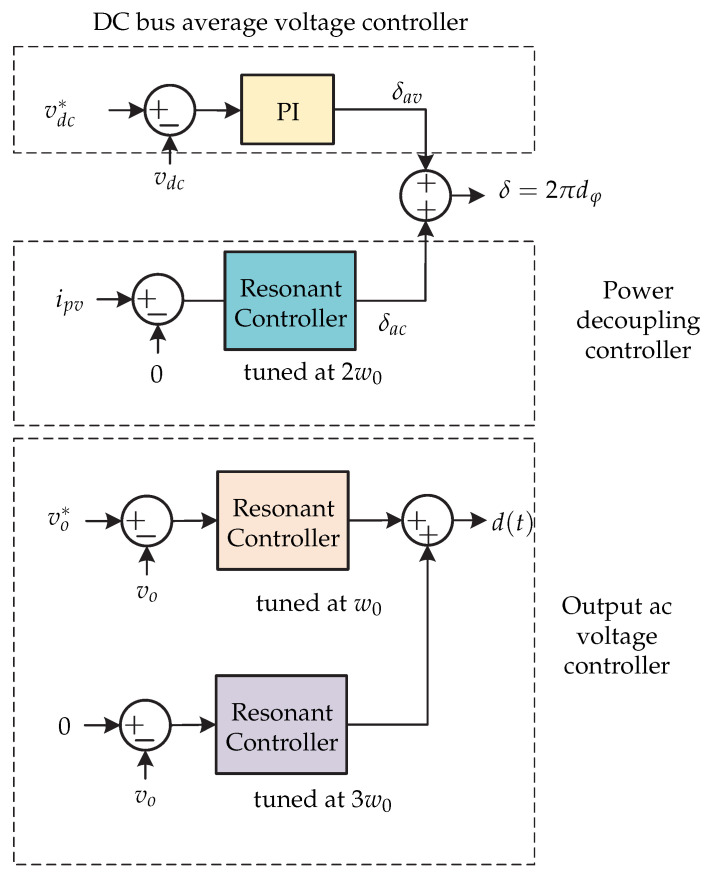
Control strategy proposed in [62].

In [62] a microinverter is presented and it consists of a high frequency dc-ac converter based on a dual active bridge (DAB) operating with zero-voltage-switching (ZVS) (Figure 22). The control strategy is presented in Figure 23 and it consists of three objectives: dc-link voltage $v_{dc}$ regulation, power decoupling, and ac output voltage $v_{o}$ regulation. First, the dc voltage is regulated by a PI controller that generates the average phase shift signal $\delta_{av}$. The result is added to the ac offset signal $\delta_{ac}$ to generate the offset signal δ. Second, a power decoupling controller generates the ac phase shift $\delta_{ac}$. It consists of a resonant controller to eliminate oscillations. Finally, two resonant drivers regulate the output ac voltage $v_{o}$ and are adjusted to reject third order harmonics and to compensate for the effects of second order.

**Figure 24:**
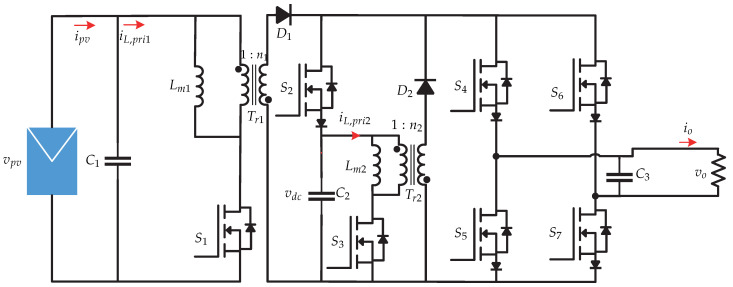
Microinverter’s topology proposed in [63].

**Figure 25:**
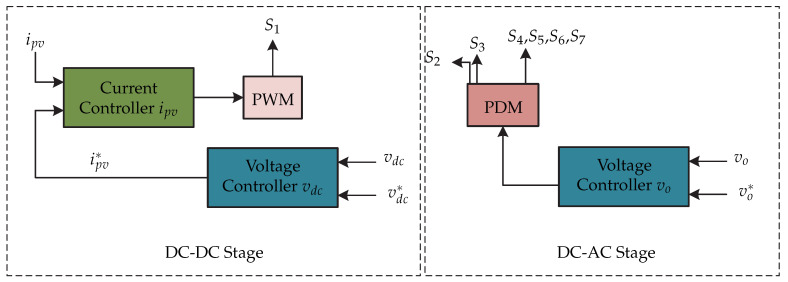
Control strategy proposed in [63].

In [63], a microinverter comprises a dc-dc flyback converter coupled to an active decoupling circuit and a full-bridge inverter (Figure 24). This can operate in island mode and it is modulated by the pulse density (PDM). Its advantages are: use of electrolytic capacitors of low magnitude and it can operate in soft switching frequency. The proposed control strategy is presented in Figure 25 and is implemented in a Field Programmable Gate Array. The strategy consists of two control loops that control the dc-dc converter and control the full-bridge inverter. The control of the dc-dc stage consists of a voltage controller of the decoupling circuit that generates a input current reference $i_{pv}^{*}$ which is compared with the current of the photovoltaic panel $i_{pv}$. The current controller generates the modulator for the switching sequence. Both controllers mentioned above are PI. On the other hand, the control of the dc-ac stage consists of a voltage controller whose input signal is the comparison of the load voltage $v_{o}$ with the reference voltage $v_{o}^{*}$. The controller output is processed by the pulse density modulation generator and generates the pulse sequence for the decoupling circuit and for the single-phase voltage source inverter.

### 3.3. Reactive Power Compensation

**Figure 26:**
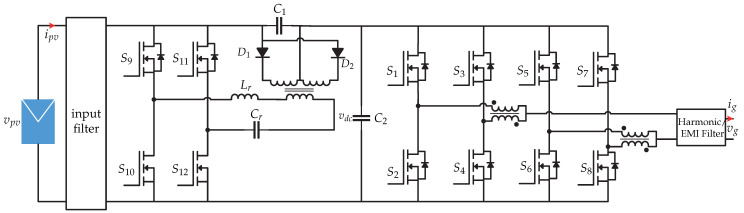
Microinverter’s topology proposed in [64].

**Figure 27:**
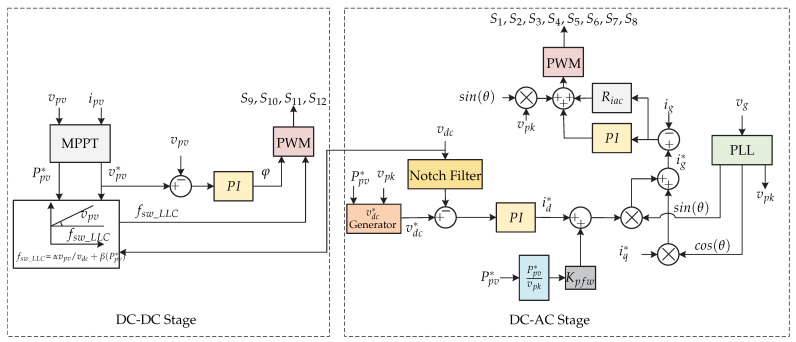
Control strategy proposed in [64].

In [64], a microinverter is introduced and provides Volt/VAR support to the power grid. The microinverter consists of a first stage with a partial power LLC resonant converter followed by an interleaved full-bridge inverter (Figure 26). The control strategy is presented in Figure 27. First, there is an MPPT algorithm to extract the maximum power from the photovoltaic panel and generate the reference voltage $v_{pv}^{*}$, which is compared to the voltage of the photovoltaic panel $v_{pv}$. The error is processed by a PI controller and generates the phase-shift angle φ. In addition, to minimize the phase-shift angle, a switching frequency feedforward loop is implemented, which is generated by the voltage of the dc link $v_{dc}$, by the output power of the photovoltaic panel $P_{pv}^{*}$ and by the reference voltage of the photovoltaic panel. This allows the dc-dc converter to change its switching frequency to maintain soft switching $f_{sw\_LLC}$ as well as to obtain an appropriate gain. In the dc-ac stage there is a double loop control. The outer voltage loop consists of the dc-link voltage regulation and is compared with a dc reference voltage $v_{dc}^{*}$ obtained from a generator as a function of the output power of the photovoltaic panel and the voltage $v_{pk}$. The error is minimized by a PI controller that generates the reference current in the *d*-frame $i_{d}^{*}$. Then, a feedforward loop in terms of the output power of the photovoltaic panel and the voltage $v_{pk}$ is added to the reference current. Both currents are added in *d* and *q* frame to generate the reference current $i_{g}^{*}$. Then the reference current is compared with the grid current and the error is minimized by a PI and resonant control (RC) to generate the duty cycle of the inverter.

**Figure 28:**
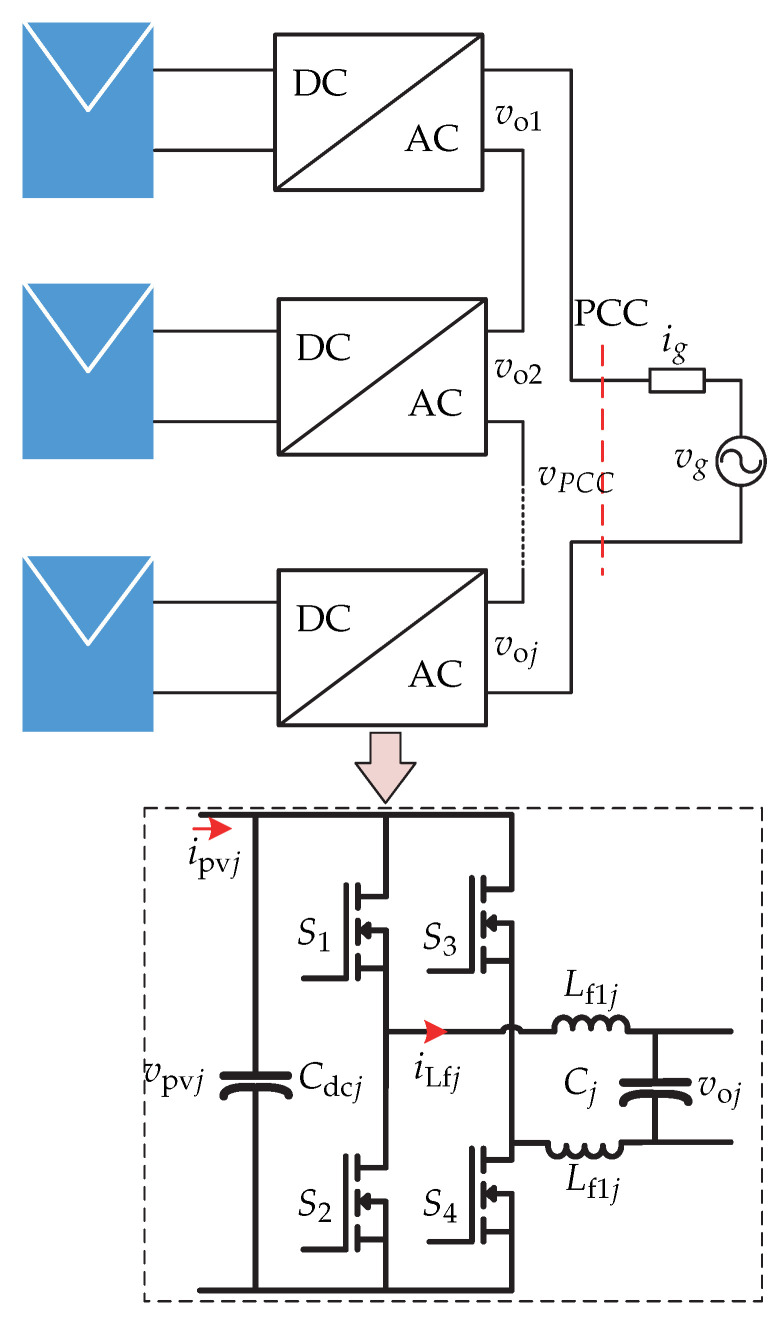
Microinverter’s topology proposed in [65].

**Figure 29:**
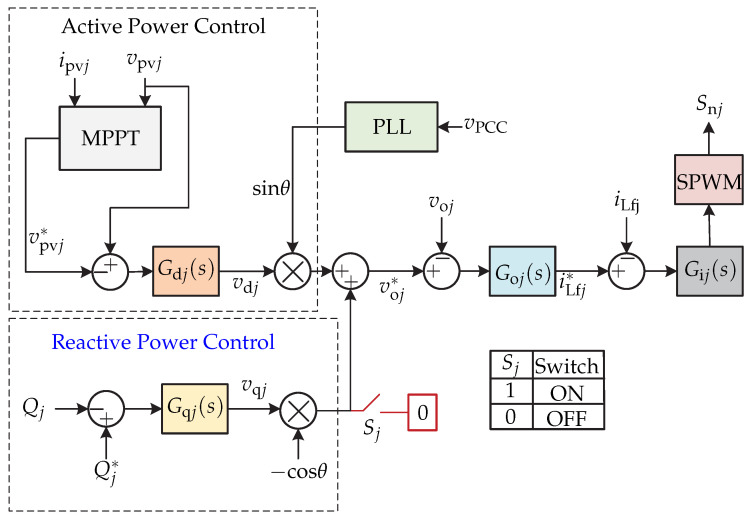
Control strategy proposed in [65].

In [65] a series configuration of microinverters consisting of cascaded full-bridge inverter is presented, where each full-bridge inverter is connected to a single photovoltaic panel (Figure 28). A distributed control strategy is proposed for each independent microinverter, in which the power is shared between the different inverters depending on the power available in the photovoltaic panels and the reactive power is controlled by a single microinverter. The control strategy is presented in Figure 29, whose objectives are: active power regulation, reactive power regulation which entails voltage, and current regulation. The control strategy first consists of an algorithm for tracking the maximum power of the photovoltaic panel to generate the reference panel voltage $v_{\mathrm{pv}j}^{*}$. The reference voltage is compared to the voltage of the sensed photovoltaic panel $v_{\mathrm{pv}j}$. The result is processed by a PI controller and generates the voltage in the *d*-frame $v_{dj}$ that is multiplied by a sinusoidal generated by a PLL of the voltage in the Point of Common Coupling. This generates the reference output voltage $v_{oj}^{*}$. The reference output voltage is compared to the sensed output voltage of the inverter $v_{oj}$ and it enters a PR controller to generate the reference inductor current $i_{Lj}^{*}$. This reference is compared to the sensed inductor current $i_{Lf}$ and it enters a PR controller to generate the modulator of the commutation sequence. The switch $S_{j}$ determines the activation of the reactive power control. The reactive power $Q_{j}$ is then compared with the reference reactive power $Q_{j}^{*}$ and the result is processed by a Proportional (P) controller and it generates the voltage of the *q* frame $v_{qj}$ multiplied by −cosθ. The result is added to the voltage in the *d*-frame to obtain the output voltage reference $v_{oj}^{*}$.

**Figure 30:**
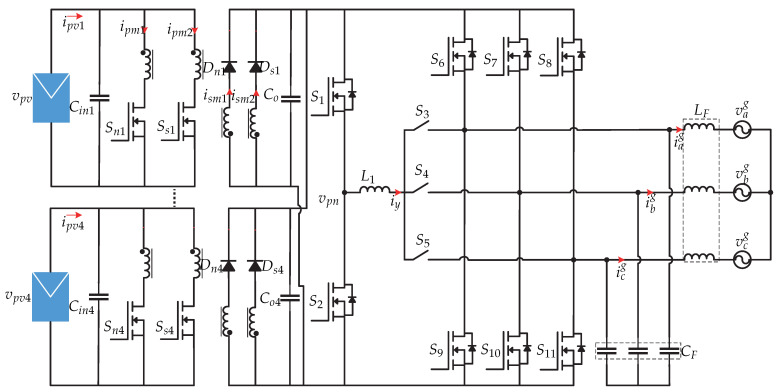
Microinverter’s topology proposed in [66].

**Figure 31:**
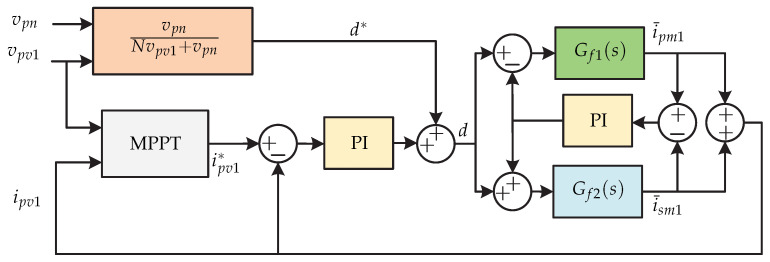
Control strategy of the MPPT proposed in [66].

**Figure 32:**
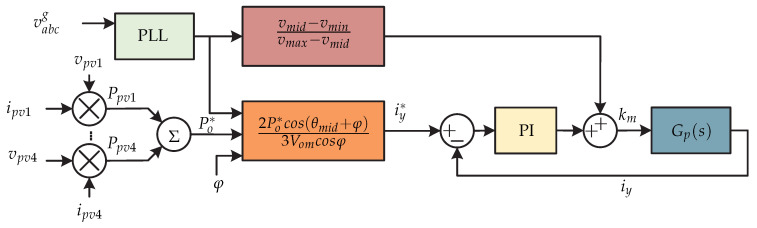
Control strategy proposed in [66].

In [66] a dual-stage microinverter without capacitors is proposed, whose dc-dc stage consists of two interleaved flyback dc-dc converters and a third harmonic current injection circuit. The dc-ac stage has a three-phase current source inverter switched at line frequency and an LC output filter (Figure 30). The flyback converter controls the MPPT and the third harmonic current injection circuit regulates the power factor correlation. In Figure 31 the control strategy for the dc-dc stage is presented. On the one hand, there is the Perturb and Observe MPPT algorithm that generates the reference input current of the flyback converter $i_{pv1}$ (outer voltage loop), and then a PI controller (inner current loop) regulates the error to generate the modulator *d*. $G_{f1}\left(s\right)$ and $G_{f2}\left(s\right)$ are the transfer functions of the first flyback converter and the second flyback converter respectively. To achieve a balance of power between the interleaved converters, a PI controller is used. The difference between the average input current of the primary flyback converter and secondary flyback converter (${\bar{i}}_{pm1}$ y ${\bar{i}}_{sm1}$) goes into a PI controller; the output is added to the duty cycle of the first flyback converter and is subtracted from the duty cycle of the second flyback converter. The photovoltaic panel current is equal to the sum of the currents of the primary flyback converter and secondary flyback converter. Figure 32 shows the control strategy of the current injection circuit and it consists of a PI current controller that compares the inductor current of the third harmonic ($i_{y}$) with a reference current $i_{y}^{*}$. The reference current is obtained by the angle of the grid voltage $\theta_{m}$ (generated by a PLL), plus the reference power $P^{*}$ (obtained by the sum of all the electrical powers of the photovoltaic panels), plus the desired output angle φ. $G_{p}\left(s\right)$ is the transfer function of the current injection circuit of the third harmonic.

**Figure 33:**
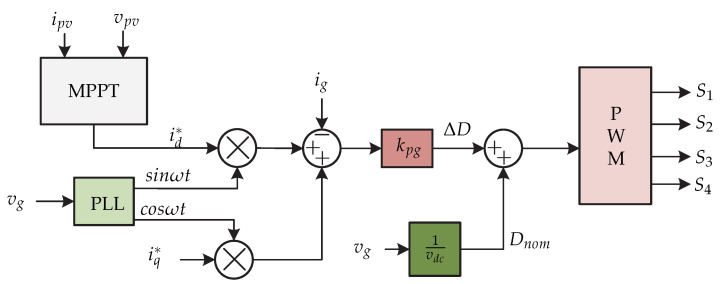
Control strategy proposed in [25].

In [25] a control strategy is presented to compensate the reactive power and is presented in Figure 33. This consists mainly of an MPPT algorithm that generates the reference current in *d* frame ($i_{d}^{*}$). Then, the currents $i_{d}^{*}$ and $i_{q}^{*}$ are multiplied by cos(ωt) and sin(ωt) respectively, whose angle is obtained by a PLL. Both reference currents are summed and compared with the grid current $i_{g}$ that multiplied by a proportional gain $k_{pg}$ generates the variation of the duty cycle ΔD. The duty cycle variation plus the nominal duty cycle generates the PWM signal.

Other control techniques are as follows:

- The paper [67] presents an active clamp flyback converter with a dual-buck inverter. The control consists of a current control (a 2-pole 2-zero compensator) for a dc-dc stage. The control technique in the dc-ac stage consists in a voltage-loop control (PI controller), a current-loop control (3-pole 3-zero compensator and feedback linearization), and a phase-locked loop. The power control is based a dq transformation.
- The paper [68] presents a two-stage microinverter and it consists of a bidirectional boost/buck dc-dc converter with coupled inductors and a full-bridge inverter. The control strategy consists of a conventional current control (PI controller) for reactive power compensation.
- The paper [69] presents a quasi Z-source (qZS) single-phase microinverter. The control strategy consists in a model predictive control with low-voltage ride-through capability.

### 3.4. Microinverter with Energy Storage

**Figure 34:**
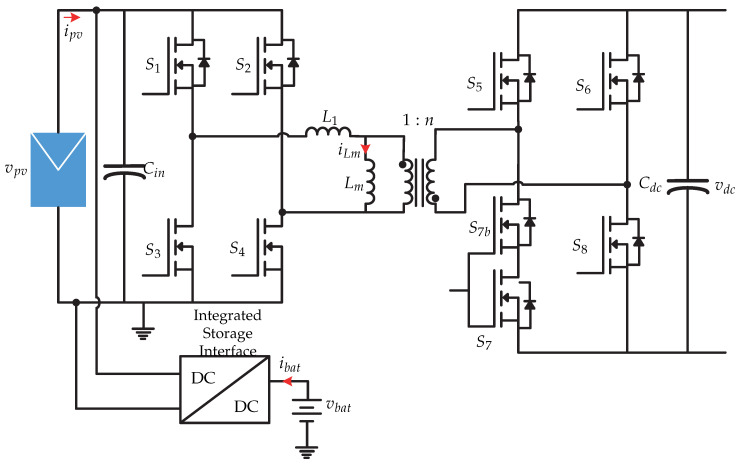
Microinverter’s topology proposed in [70].

**Figure 35:**
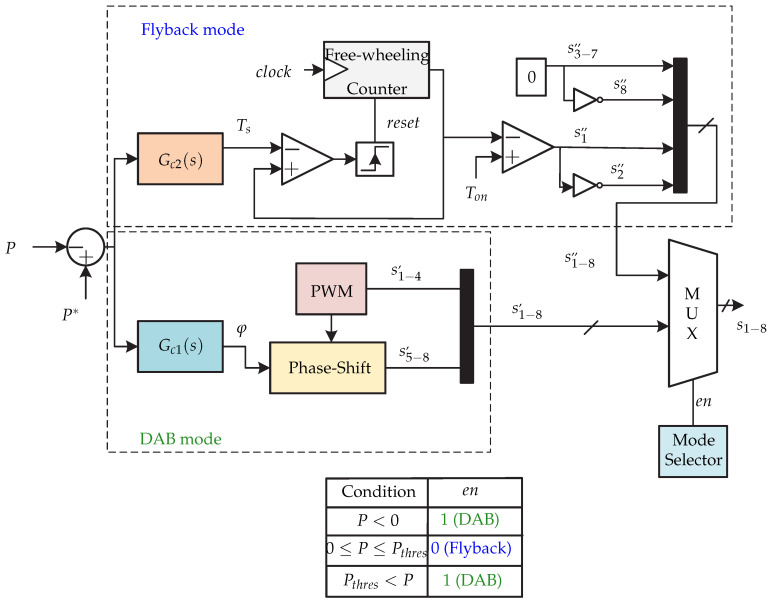
Control strategy proposed in [70].

In [70] a microinverter with integrated storage is presented and it consists of a dual active H-bridge dc-dc converter (DAB); in addition, a dc-dc converter connected to a battery is coupled in parallel (Figure 34). This work only presents the connection stage of the photovoltaic panel and the battery with dc-link. The proposed dc-dc converter has two modes: on the first hand, it works as a dual-active H-bridge (DAB) converter, and on the second hand it works as a dual-transistor flyback converter. The proposed dual control strategy is presented in Figure 35. This consists of different conditions of the power flow *P*, which depending on the condition, the flyback mode or dual active H-bridge mode is selected. The article [70] does not present more details of the control strategy, it only mentions that the comparison between the power flow calculated with the reference is processed by a controller $G_{c2}\left(s\right)$ generating $T_{s}$, to then generate the switching sequence of the flyback mode. In the case of DAB mode, the controller $G_{c1}\left(s\right)$ generates an angle ϕ to regulate the power flow tofrom the dc-ac stage. The multiplexer determines the sequence of commutation depending on the selected mode.

Other control techniques are as follows:

- The paper [71] presents a high-frequency push–pull topology with galvanic isolation with a voltage source inverter. The control technique consists of MPPT controller, a battery charge algorithm (constant current followed by constant voltage control), a dc-link voltage regulator (PI controller), and a current-loop control based a model predictive control.
- The paper [72] presents a dual-active bridge microinverter topology with integrated energy storage capability. The control strategy comprises a cascaded loop with two PI controllers and a two-loop approach with PI controller and PR controller.

### 3.5. Multi-Modes or Multiples Functions

**Figure 36:**
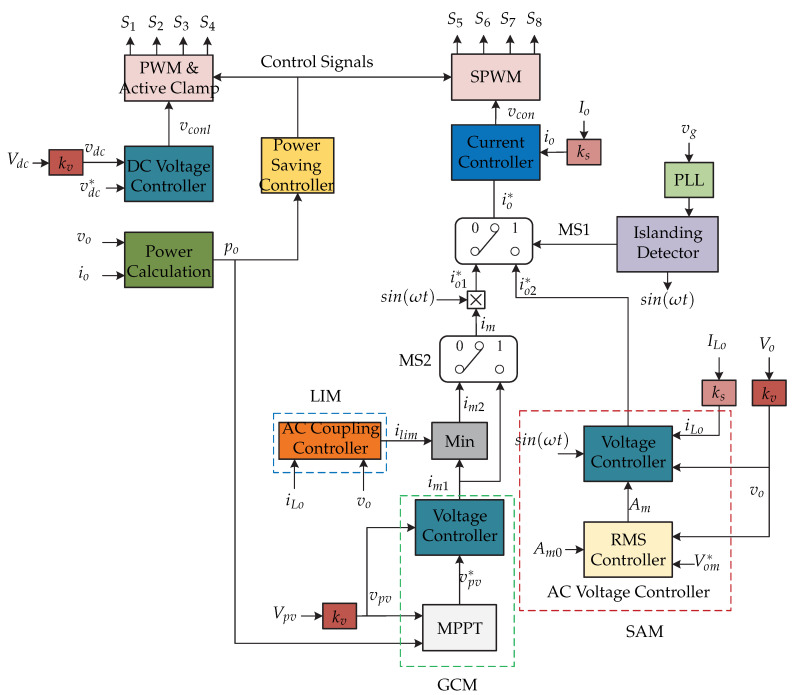
Control strategy proposed in [73].

**Figure 37:**
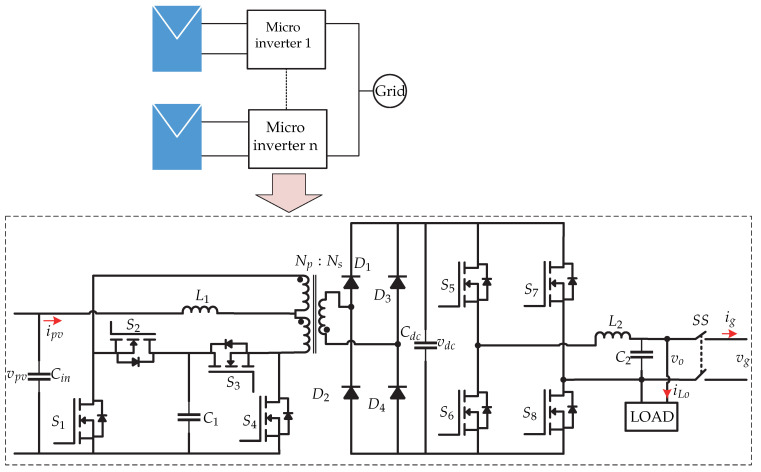
Microinverter’s topology proposed in [73].

In [73], two-stage topology of microinverters is presented and the multi-mode control strategy is presented in Figure 36. The first stage is a active-clamped current-fed push–pull converter and the second stage is an full-bridge inverter. These microinverters are connected to each other in cascade, between the grid and the load Figure 37). It has three operation modes: (1) grid-connected mode (GCM), (2) line-interactive mode (LIM), and (3) stand-alone mode (SAM). The multi-mode control strategy is presented in Figure 36. First, the dc-dc converter regulates the dc-link voltage $v_{dc}$ by means of a PI type voltage controller. The full-bridge inverter is commanded by the output current control $i_{o}$, whose reference is generated from the GCM, SAM, or LIM modes. The island detector allows to select the grid-connected mode or off-grid mode through MS1. In the event of a power grid failure, the SS switch opens and the island detector selects SAM mode. In SAM mode, a controller regulates the ac voltage of the inverter $v_{o}$ and it generates the reference current $i_{o2}^{*}$. In the case that the grid voltage is maintained under normal conditions, the SS switch remains closed; therefore, the microinverter is connected to the grid. In this case, there are two modes of operation, the GCM or the LIM. They are selected by the MS2 signal in order to determine the reference current amplitude $i_{m1}$ o $i_{m2}$. In the case of GCM mode, an MPPT algorithm is used to inject the maximum available power from the photovoltaic panel into the grid. The MPPT algorithm generates the voltage reference of the photovoltaic panel $v_{pv}^{*}$ and it is compared to the sensed voltage panel $v_{pv}$, which then through a voltage controller generates the reference current $i_{m1}$. In LIM mode, the reference current $i_{m2}$ is generated by the minimum value of the voltage controller of the photovoltaic panel and an ac coupling controller, whose input signals are the output voltage $v_{o}$ and the load current $i_{Lo}$.

**Figure 38:**
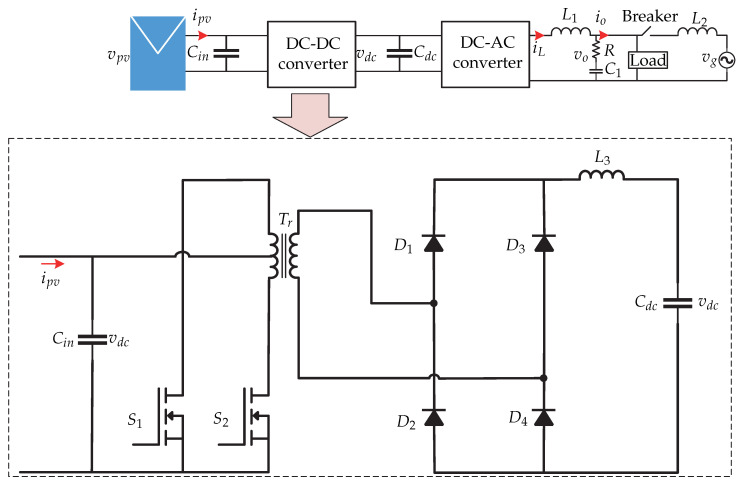
Microinverter’s topology proposed in [74,75,76].

**Figure 39:**
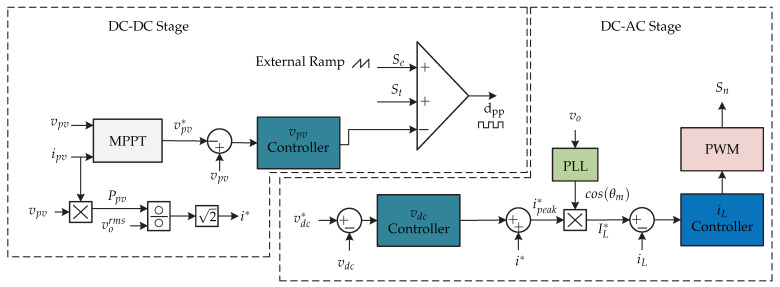
Control strategy proposed in [74,75,76].

In [74,75,76] a dual-stage microinverter is presented and it consists of a push–pull dc-dc converter connected to a full-bridge inverter (Figure 38). The proposal of the article is a microinverter that can operate both in island mode and in mode connected to the electrical grid without the need to modify the control algorithm. In grid-connected mode, the microinverter must inject electrical power; on the other hand, in island mode, the microinverter must deliver an appropriate ac voltage to local loads. The control strategy of Figure 39 for the grid-connected mode consists of an internal loop that controls the injection of current $i_{L}$, while an external loop controls the voltage of the dc-link $v_{dc}$. In this case, the push–pull converter controls the MPPT algorithm, whose reference voltage $v_{pv}^{*}$ is compared with the voltage of the photovoltaic panel $v_{pv}$ that is processed by a voltage controller. In addition, the proposed control in the dc-dc stage limits the transformer current to avoid its saturation.

**Figure 40:**
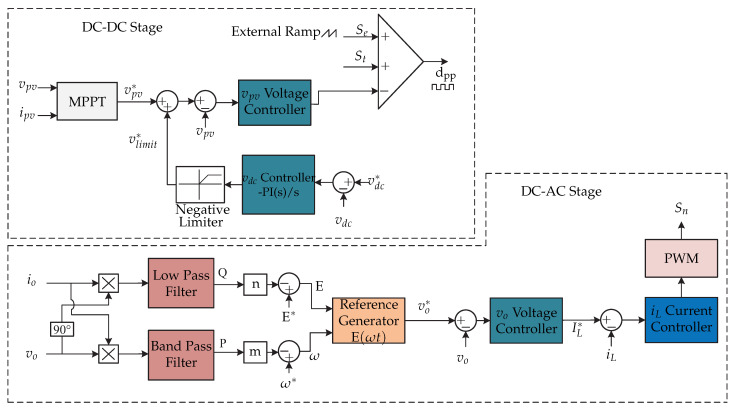
Control strategy proposed in [74,75].

On the other hand, in Figure 40, the control strategy in island mode is presented. In island mode, the inner current loop does not change with respect to the aforementioned, but the outer voltage loop regulates the output voltage $v_{o}$ (voltage source control algorithm). This is compared to a reference voltage $v_{o}^{*}$, generated by a droop control as a function of active and reactive power. The control of the dc-dc stage controls the voltage in the dc link by means of a voltage controller generating the limit of the reference voltage and is added to the reference voltage of the photovoltaic panel. All voltage controllers are PI, while current controllers are PR. The characteristic of the proposed reconfigurable control is that there are no transients between the microinverter and the load when switching from one mode to another.

Other control techniques are as follows:

- The paper [77] presents a two-stage microinverter and it consists of dc-dc triple active bridge (TAB) converter that integrates back-up battery; and the second stage is a voltage source inverter (VSI) that operates in both grid-connected mode (GCM) and stand-alone mode (SAM). The control algorithm consists in a central control based in a mode transition scheme. Each mode has PI controllers to regulate the current grid, current load, and dc-link voltage; it has a PR controller to regulate the load voltage.
- The paper [78] presents a buck-boost dc-dc converter cascaded interleaved flyback dc-dc converter with a unfolding bridge inverter. The control technique consists in a droop control and a peak current control.
- The paper [79] presents a current-fed push–pull, full-wave rectifier with full-bridge. The microinverter can operate in island mode and grid mode. The control technique comprises of different PI controllers for both modes.

## 4. Discussion

Table 1 presents the characteristics of the revised control strategies, in order to make a comparison between them. Table 1 shows the stability results as a function of the gain margin (GM) and phase margin (PM). In addition, the quality of the signals is presented as a function of the total harmonic distortion (THD) and the dynamic responses are also presented as a function of the settling time ($t_{s}$) in millisecond and the overshoot ($M_{p}$). In addition, the efficiency of the maximum power point tracking algorithm (MPPT) is presented.

Most of the authors use a PI controllers because they are simple and easy to design. As the microinverters are non-linear systems, the PI control is capable of working properly only in a reduced operating region, this being a disadvantage of the controller. In addition, it is necessary to determine the gain values of the controller, which is sensitive to variations and uncertainty of the microinverter parameters [80]. In addition, the revised articles tend to use the PR control that has better performance in the regulation of alternating current as compared to the PI control [81]; however, it also has the problem of sensitivity to variations and uncertainties. From this point of view, a feasible solution is to implement other control techniques, such as model-based predictive control [82], feedback linearization control [83], and fuzzy logic control [84].

In addition, as can be seen in Table 1, the different characteristics depend on both the topology (design) and the control strategy. The design determines the values of the various components contained in the microinverter to meet voltage and current requirements and also determines the size of the microinverter. In addition, this determines the voltage and current ratings of the power semiconductors. The selection of electronic components will mainly influence the efficiency of the microinverter. Finally, once the topology is designed and the components are selected, it is necessary to regulate the voltage and current levels despite the presence of disturbances. Therefore, it is necessary to implement control techniques that ensure a correct operation of the microinverter to meet the different voltage and current requirements and thereby increase the reliability and robustness of the microinverter.

## 5. Conclusions

This article presented an overview of microinverters and a review of control strategies for applications such as grid-connected, island mode, reactive power compensation, incorporation of energy storage, and multi-modes. Microinverters are a promising solution to mitigate the problems of using photovoltaic panels, such as partial shading. Within the reviews it can be inferred that there is an increase of studies on microinverters, due to the advancement of semiconductor technology as well as cost reduction. From the point of view of the control strategy, research tends to use PI controllers and PR.

The control strategies were studied for the different applications in microinverters. The proposed control strategy was described as well as the associated power converters. It can be mentioned that due to the advancement of microprocessors, it is tending to the incorporation of multiple controls and multiple modes in the microinverters, increasing reliability and functionality of the photovoltaic system.

Finally, it can be mentioned that although the design and selection of the electronic components in a power converter topology is important and essential in the conversion efficiency, the design of the control strategy is a third important factor in increasing the reliability and functionality of microinverters.

## Figures and Tables

**Table 1:** Characteristics of the revised control strategies.

Cite	GM (dB)	PM	THD (%)	$t_{s}$ (ms)	$M_{p}$	MPPT (%)	Advantage	Topology	Controller
[12]	-	-	4.37($i_{g}$)	-	-	-	Low frequency Harmonic mitigation. Switching losses reduction.	Full bridge inverter	Hysteresis + PR
[13]	-	-	2.87($i_{g}$) 3.09($v_{g}$)	20($i_{g}$)	-	99.7	High reliability Wide operating range. Fast MPPT.	Boost-half-bridge converter + full-bridge inverter	PI controllers + Resonant control
[15]	12($i_{g}$) inf($v_{dc}$)	58°($i_{g}$) 74°($v_{dc}$)	2.5($i_{g}$)	-	-	-	Fast dynamic response.	LLC resonant converter + 3-phase dc-ac converter	PI Controllers
[18]	inf($i_{L2}$)	85.9°($i_{L2}$)	3.18($i_{g}$)	-	-	96	High reliability. Reactive power support. LVRT Capacity.	Non-inverted Cuk connected to an inverter Cuk converter	PI and PR controllers
[19]	-	-	-	-	-	98.5	Ability to estimate current for different inductance values. Elimination of measurement noise.	Flyback converter + VSI	PI control + PCM + State Observer
[21]	-	-	Off-grid 0.5($v_{g}$) On-grid 2.4($i_{g}$)	-	-	-	LVRT and anti-island capability.	Flyback converter + VSI	PI controllers + DQ power estimation
[25]	-	-	3.8($i_{g}$)	-	-	-	High conversion efficiency High reliability	Flyback converter + series resonant voltage doubler	P controller + feedback linearization + Voltage controller
[26]	-	-	$3(i_{g})$	-	-	-	Simple. Elimination of distortion caused by zero crossing.	Coupled-inductor double-boost inverter	PI controllers
[27]	-	-	-	10($v_{o}$)	$5V(v_{o})$	-	Switching losses reduction.	DMR based series resonant dc-dc converter	PI controllers
[62]	-	-	0	70($v_{dc}$)	-	-	ZVS capacity. Switching losses reduction. Decoupling capacitance reduction.	Flyback converter + VSI	PR control + HC
[29]	inf($i_{o}$)	45°($i_{o}$)	2.4($i_{o}$)	-	-	-	Fast dynamic response Harmonic frequencies elimination. Low computational burden. Elimination of disturbances. Hybrid operation(DCM-CCM).	DAB inverter	PI + RC controllers
[63]	-	60°($i_{pv}$) 60°($v_{dc}$) 60°($v_{o}$)	3.73($i_{o}$)	1000($v_{dc}$)	-	-	ZCS switching capacity. Wide range of input voltages.	Flyback + active decoupling circuit + full-bridge inverter	PI controllers
[64]	-	-	-	-	-	-	Reactive power support. High Efficiency	LLC resonant converter + interleaved full-bridge inverter	PI controllers + RC + feedforward loop
[65]	-	-	-	-	-	-	Distributed control. Active and reactive power control capacity.	Cascaded full-bridge inverter	Distributed control: P + PI + PR controllers
[66]	-	-	Full load 4.29($i_{o}$) Partial load 6.82($i_{o}$)	-	-	-	Controllable power factor. High efficiency. Independent MPPT control.	Interleaved flyback converter + 3-phase Current Source Inverter	PI controllers
[70]	-	-	-	0.04($i_{pv}$)	0.2 A($i_{pv}$)	-	Two modes of operation Flyback and DAB. Reduction of switching losses. High stability.	DAB + dc-dc converter for the batteries	Dual-mode control
[73]	-	-	-	-	-	-	Multifunctionality. Parallel multi-mode operation.	Push–pull converter + full-bridge inverter	PI controllers
[74,75]	-	On-grid 89.6°($v_{pv}$) 58.6°($i_{g}$) 87°($v_{dc}$) Off-grid 90.2°($v_{pv}$) 65.2°($v_{o}$) 76.2°($v_{dc}$) 81.2°($i_{o}$)	0.05 s($v_{g}$)	-	-	-	Ability to operate off-grid and on-grid. Reconfigurable Control.	Push–pull converter + full-bridge inverter	PI + PR controllers

## Data Availability

Not applicable.

## Abbreviations

The main abbreviations and nomenclatures that are used in this manuscript are listed below:

ACC	Phase Accumulator
DAB	Dual active bridge
DMR	Dual Mode Rectifier
CVT	Constant Voltage Method
EMI	Electromagnetic Interference
GCM	Grid Connected Mode
GM	Gain Margin
HC	Harmonic Compensator
LIM	Line-interactive Mode
PCM	Peak Current Control
PDM	Pulse Density Modulator
PM	Phase Margin
SAM	Standalone Mode
RC	Repetitive Controller
THD	Total Harmonic Distortion
ZVS	Zero Voltage Switching
$A_{m}$	Voltage amplitude reference
$C_{dc}$	DC-link capacitor
$C_{F}$	Filter capacitor
$C_{in}$	Input capacitor
$C_{x}$	Capacitor x=1,2…
$C_{o}$	Output capacitor
$C_{r}$	Resonant capacitor
*d*	Duty cycle
$D_{CCM}$	Duty cycle in continuous conduction mode
$D_{DCM}$	Duty cycle in discontinuous conduction mode
$D_{n}$	Diode *n*, where n=1,2⋯
$D_{nj}$	Diode *n* of the microinverter j=1,2⋯
$D_{nom}$	Rated duty cycle
$D_{p}$	Hybrid nominal duty ratio
$d_{pp}$	Switching signal pattern
*E*	Amplitude of the inverter voltage
en	Enable the mode selector
$f^{*}$	Reference of switching frequency
$f_{o}$	Sampling frequency
$f_{s}$	Switching frequency
G(s)	Transfer function
$i_{bat}$	Battery current
$i_{Cn}$	Current of capacitor n=1,2,3⋯
$i_{d}$	*d* Frame current
$i_{g}\left(t\right)$	Grid current
$I_{g}^{*}$	Amplitude of the grid current reference
$i_{L}$	Inductor current
$i_{lo}$	Load current
$i_{L,pri}$	Current of the primary side transformer
$i_{L,sec}$	Current of the secondary side transformer
$i_{lim}$	Current amplitude limit reference
$i_{pv}$	Photovoltaic panel current
${\hat{I}}_{pv}$	Estimated photovoltaic panel current
$i_{o}$	Output current
$i_{q}$	*q* Frame current
*k*	Sensor gain
$K_{d}$	Derivative gain
$k_{m}$	Duty ratio
$k_{pg}$	Proportional gain
$L_{F}$	Filter inductor
$L_{in}$	Input inductor
$L_{m}$	Magnetizing inductor
$L_{n}$	Inductor *n*, where n=1,2,3⋯
$L_{o}n$	Output inductor *n*, where n=1,2⋯
$L_{s}$	Inductor of transformer
$L_{r}$	Resonant inductor
*m*	Amplitude droop coefficient
$m_{x}$	Modulator signal x=a,b,c
$M_{p}$	Overshoot
*n*	Frequency droop coefficient
$p_{o}$	Output power of the inverter
$P^{*}$	Power reference
*P*	Active power
$P_{pv}$	Photovoltaic panel power
*Q*	Reactive power
*R*	Resistor
$s_{1-8}$	Gating voltages for switches $S_{1-8}$
$S_{n}$	Switch *n*, where n=1,2,3⋯
$T_{on}$	Fixed on-time
$t_{s}$	Settling time
$T_{s}$	Switching period
$v_{ab}$	Inverter voltage
$v_{bat}$	Battery voltage
$v_{cd}$	Voltage between the terminals cd
$v_{d}$	Voltage in the *d* frame
$v_{dc}$	DC-link voltage
$v_{Cn}$	Voltage of capacitor n=1,2,3⋯
$v_{g}$	Grid voltage
$v_{mpp}$	Voltage in the maximum power point
$v_{max}$	Maximum value of the dc-link voltage
$v_{mid}$	Medium value of the dc-link voltage
$v_{min}$	Minimum value of the dc-link voltage
$v_{limit}^{*}$	Limit voltage reference
$v_{o}$	Output voltage
$V_{om}$	Magnitude of the output phase voltage.
$v_{PCC}$	Voltage in the point common coupling
$v_{pv}$	Photovoltaic panel voltage
$v_{q}$	Voltage in the *q* frame
Δ	Disturbance or variation
φ	Output angle
δ	Offset signal
$\theta_{m}$	Angle of the grid voltage
ω	Frequency of the inverter voltage
